# Anabolic steroids as the substrate for atrial fibrillation: a case report

**DOI:** 10.1093/ehjcr/ytae585

**Published:** 2024-10-28

**Authors:** Manal Yebari, Abderrahmane Bouchaala, Issam Berraj

**Affiliations:** Emergency Department, Ibn Sina Hospital, Rabat, Morocco; Emergency Department, Ibn Sina Hospital, Rabat, Morocco; Emergency Department, Ibn Sina Hospital, Rabat, Morocco

**Keywords:** Atrial fibrillation, Anabolic steroids, Arrhythmia, Case report

## Abstract

**Background:**

Atrial fibrillation (AF) is the most frequently encountered sustained arrhythmia worldwide. This supraventricular rhythm disorder is precipitated by advanced age, valvular heart disease, hypertension, heart failure, congenital heart defects, and others. However, the role of anabolic steroids (ASs) abuse in precipitating AF remains insufficiently researched and largely underreported, despite their known cardiovascular risks.

**Case summary:**

We present the case of a 40-year-old male bodybuilder who was admitted to the emergency department with symptomatic AF. His medical history revealed the use of ASs, which was suspected to be the trigger. A thorough biological evaluation and echocardiography were performed, revealing no structural or functional cardiac abnormalities. After electrical cardioversion and discontinuation of AS use, the patient’s rhythm returned to normal, with no recurrence of arrhythmia during follow-up.

**Discussion:**

This case highlights the potential but underexplored link between AS use and the onset of AF. Although ASs are known to affect cardiovascular health by promoting hypertension, left ventricular hypertrophy, and endothelial dysfunction, their role in arrhythmogenesis, particularly in AF, remains unclear. In this patient, the absence of other identifiable triggers, combined with the resolution of symptoms upon steroid cessation, strongly suggests a causal relationship. Further research is needed to clarify the mechanisms through which ASs may contribute to the development of AF, particularly in younger, otherwise healthy individuals such as athletes and bodybuilders. This case underscores the importance of awareness among clinicians regarding the potential cardiac risks associated with AS use.

**Learning points**

- Awareness of anabolic steroids (ASs) as a risk factor: This case emphasizes the need for clinicians to consider AS use as a potential risk factor for atrial fibrillation (AF), especially in patients without traditional cardiovascular risk factors.
- Reversibility of AF: The case demonstrates that discontinuation of ASs can lead to the resolution of AF, suggesting the importance of patient education and lifestyle modification in managing this condition.

## Introduction

Atrial fibrillation (AF) is the most frequently encountered sustained arrhythmia worldwide, affecting an estimated 59.7 million individuals globally as of 2019.^1^ Well-documented triggers include advanced age, hypertension, valvular heart disease, and heart failure.^2,3^ Despite extensive research on these traditional risk factors, the role of anabolic steroids (ASs) in precipitating AF remains poorly understood and underreported.^4^

This case is unique because it highlights a rare but significant association between AS abuse and the onset of AF in a young, otherwise healthy individual.

## Summary figure

**Figure:**
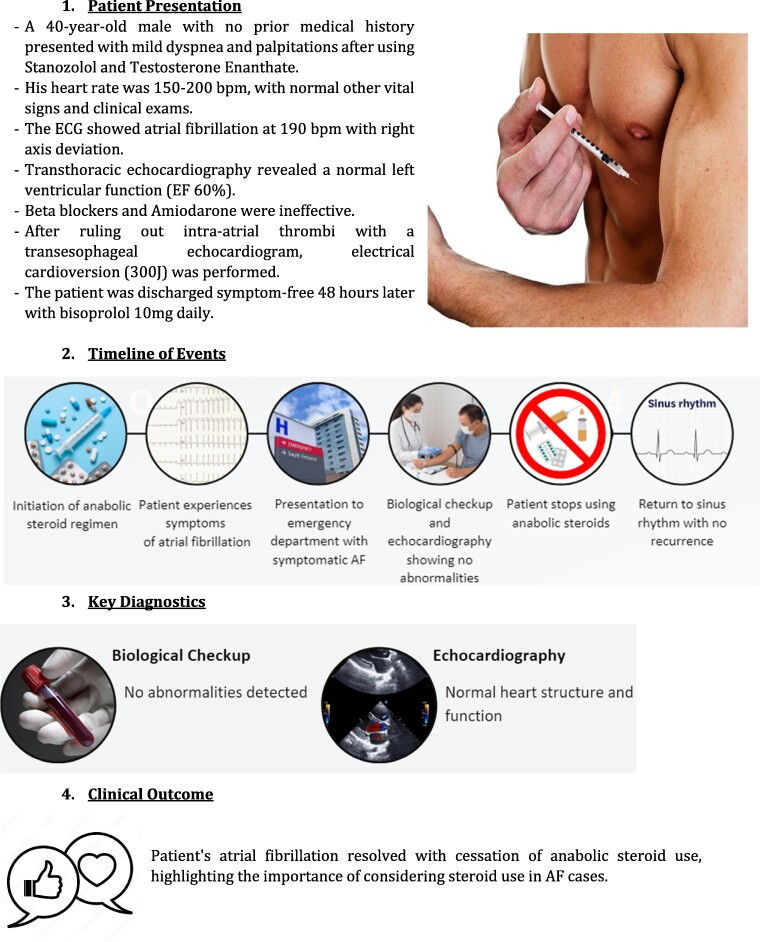


## Case presentation

A 40-year-old Moroccan male patient, with a history of alcohol use without smoking or any previous medical history, presented to the emergency department with mild dyspnoea (NYHA class II) of progressive onset and palpitations, without any episodes of syncope, or chest pain. The patient is a professional bouncer with over 20 years of training experience. He follows a regimen of four weekly sessions, consisting of 2 days of aerobic conditioning, such as treadmill running and stationary cycling, and 2 days of resistance training. Each session lasts ∼60 min at moderate to high intensity. After thorough questioning, the patient confirmed taking oral stanozolol 10 mg daily and intramuscularly 250 mg testosterone twice a week for 1 month before the onset of his symptoms. He was assessed for chronic obstructive pulmonary disease and obstructive sleep apnoea syndrome. A Pittsburgh Sleep Quality Index (PSQI) questionnaire was completed, with results suggesting normal sleep patterns. Additionally, a pulmonary function test showed a forced expiratory volume in 1 s (FEV1) at 95% of predicted value, indicating no significant evidence of chronic obstructive pulmonary disease.

The patient has a body mass index of 29.2 and a waist circumference of 92 cm. His vital signs were as follows: afebrile (37.1°C), blood pressure of 130/70 mmHg, and a heart rate oscillating between 150 and 200 b.p.m., respiratory rate of 19 breaths/min, and oxygen saturation of 97% on room air. The clinical examination revealed an irregular heartbeat upon cardiac auscultation and acne, which could be indicative of AS abuse.^5^

**Figure 1:**
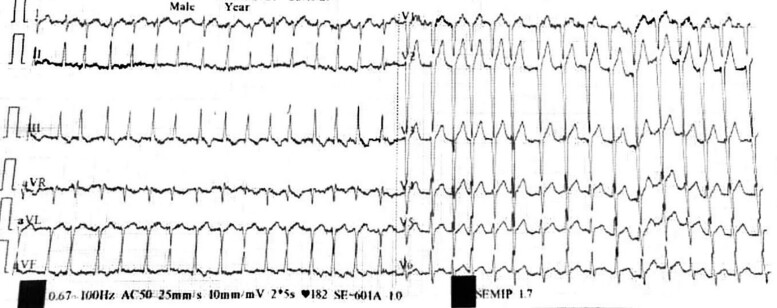
Pre-electrical cardioversion electrocardiogram.

On chest X-ray, there was mild bilateral hilar overload. Biological check-up, including natremia, kalemia, urea, creatinine, liver function enzymes, blood sugar, hypersensitive cardiac troponin I, TSH, T3, and T4, was normal (*Table 1*). The electrocardiogram (ECG) (*Figure 1*) showed AF at 190 b.p.m. with a right axis deviation; negative T waves in II, III, and AVF leads; and poor R wave progression in precordial leads.

**Table 1:** Blood test results

Parameter	Result
Ggt (gamma-GT)	30 U/L
HDLc (HDL)	50 mg/dL
LDLc (LDL)	90 mg/dL
Hct (haematocrit)	45%
TSH (thyroid-stimulating hormone)	2.5 mUI/mL
K (serum potassium)	4.2 mmol/L
Mg (serum magnesium)	2.0 mg/dL

**Figure 2:**
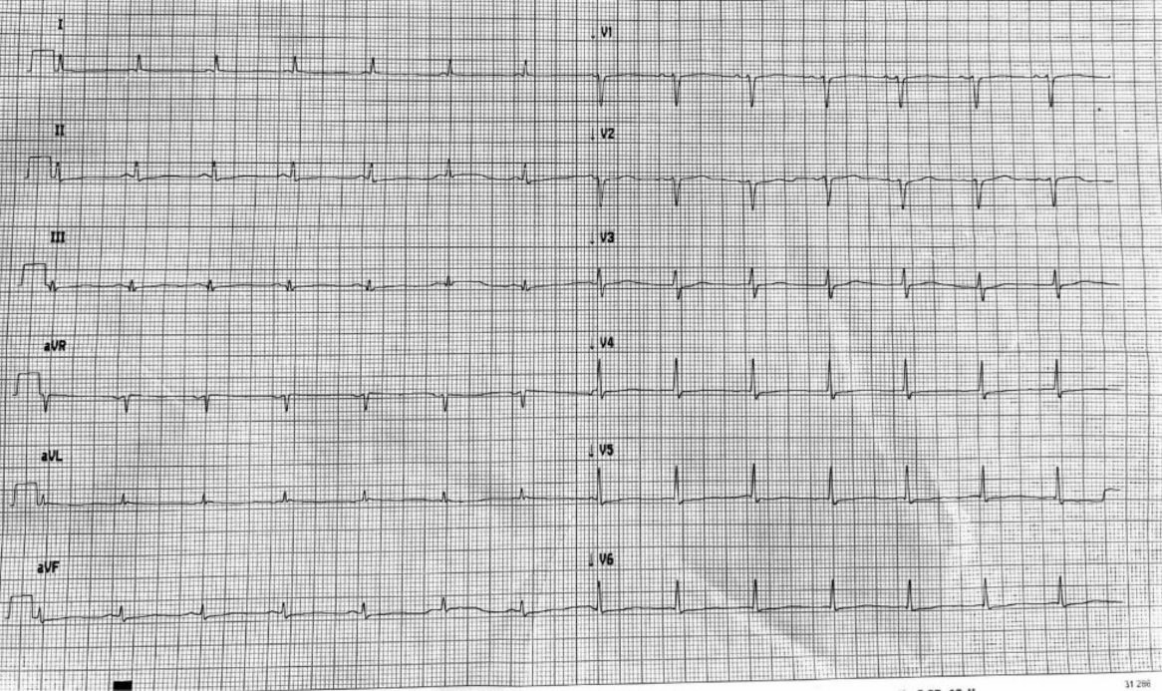
Post-electrical cardioversion electrocardiogram.

The patient was initially started on a beta-blocker, without symptomatology improvement which justified use of amiodarone, with no significant result. Transoesophageal echocardiogram was performed to formally exclude intra-atrial thrombi, and then, the patient underwent an electrical cardioversion (300J) with return to sinus rhythm. The post-cardioversion ECG (*Figure 2*) showed no repolarization abnormalities, and subsequent ECGs performed in the following days revealed no ST-T changes.

Transthoracic echocardiography (TTE) found a non-dilated, non-hypertrophic left ventricle with conserved global and segmental contractility, a left ventricular ejection fraction (LVEF = 60%), an E wave at 0.9 m/s, an A wave at 0.6 m/s, an E′ at 0.9 m/s, and estimated pulmonary arterial systolic pressure (PASP = 30 mmHg), with the absence of any valvular heart disease, intracavitary thrombi or spontaneous contrast, cavitary dilatations, or other structural abnormalities.

The patient was discharged 48 h later after complete remission of symptoms, with bisoprolol 10 mg daily. After discontinuing AS use, there was no recurrence of arrhythmia during follow-up.

## Discussion

Atrial fibrillation is the most frequently encountered sustained arrhythmia worldwide.^1,6^ The number of new AF cases is doubling every few decades. This rise in prevalence is primarily due to factors such as population growth, aging, and improved survival rates from other cardiac conditions.^7,8^ Although the majority of patients presenting with AF are found to have an underlying risk factor such as hypertension, diabetes, sleep apnoea syndrome, obesity, heart failure, alcohol consumption, physical activity, and endocrine or metabolic disorder, in about 15% of cases, no definitive diagnosis is ever established.^6,9^ In the younger population, paroxysmal AF is the most frequent form, yet its prevalence remains poorly elucidated.^10,11^

Anabolic steroid abuse has been linked to dysfunction in various organ systems, especially the cardiovascular system including hypertension, thromboembolic events, ventricular hypertrophy, and acute myocardial infarction through the acceleration of coronary atherosclerosis.^12–19^ Yet, AF related to the abuse of ASs remains poorly reported, with only two case reports, with doses that are significantly higher than the therapeutic dosage of AS: one involving moderate left atrial hypertrophy^20^ and the other with a structurally normal heart.^21^ According to some studies, the plausible arrhythmogenic mechanisms of ASs are myocardial structural and molecular remodelling and autonomic dysfunction.^22,23^ Also, the use of large doses of ASs is associated with increased QT dispersion despite short QT intervals, which possibly reflects altered myocardial structure in the hypertrophied heart and increases the risk of malignant arrhythmias.^24^

Our patient is a bodybuilder and an alcohol consumer, both of which are well-established risk factors for AF. Moreover, it should be acknowledged that a genetic predisposition for AF cannot be excluded. Therefore, while AS use may contribute to the development of AF, it is essential to consider these additional risk factors in the patient’s overall assessment.

Regarding AS abuse, the patient had been taking high doses for 1 month. Clinically, the only sign of AS abuse was acne, without other typical manifestations. Biologically, the patient’s laboratory tests were surprisingly normal, with no major abnormalities in liver enzymes or lipid profiles, suggesting that the effects of steroids can vary significantly among individuals.

On ECG, the patient showed ST-T changes, which could indicate repolarization abnormalities potentially linked to either ischaemia or steroid abuse. However, after cardioversion, these changes resolved, suggesting that the abnormalities were likely secondary to the arrhythmia itself rather than underlying ischaemic heart disease. Given the absence of angina symptoms and the normal findings on TTE, we did not pursue further coronary artery disease evaluation.

Finally, while many studies have explored the link between ASs and AF, establishing a direct cause-and-effect relationship remains challenging.^25^ The association is often speculative and serves as a diagnosis of exclusion, particularly when other competing risk factors, such as alcohol use and intense physical activity, are present. More research is needed to better understand the mechanisms through which ASs may contribute to arrhythmias like AF.

## Conclusion

This case highlights the potential role of AS abuse as a contributing factor to the AF in a young bodybuilder. Although the current literature does not provide strong evidence for causality between AF and AS abuse, the increase in the use of these substances among young athletes, coupled with multiple reports of AF in this population, suggests a potential causal link.

## Data Availability

No new data were created or analysed in this study.

## Lead author biography

Dr Manal Yebari is a cardiology resident at the University Hospital Center Ibn Sina in Rabat. She holds a medical degree from the Faculty of Medicine and Pharmacy of Rabat. Dr Manal Yebari is passionate about arrhythmias and sports cardiology, focusing on the cardiovascular effects of performance-enhancing substances. Through her clinical practice and research, she aims to improve the understanding and management of heart rhythm disorders, especially in young, athletic populations. Dedicated to advancing cardiovascular health, she is committed to evidence-based medicine and patient education.

## Notes

**Consent:** The authors confirm that written consent for publication of this case report was obtained from the patient in accordance with the COPE guidelines.

**Funding:** None declared.
